# Social Jetlag and Its Association With Screen Time and Nighttime Texting Among Adolescents in Sweden: A Cross-Sectional Study

**DOI:** 10.3389/fnins.2020.00122

**Published:** 2020-02-18

**Authors:** Momota Hena, Pernilla Garmy

**Affiliations:** ^1^Department of Health Sciences, Faculty of Medicine, Lund University, Lund, Sweden; ^2^Faculty of Health Sciences, Kristianstad University, Kristianstad, Sweden

**Keywords:** sleep, screen time, nighttime texting, adolescents, social jetlag

## Abstract

The discrepancy between social and biological clock due to sleep and wake up time difference across weekdays and weekends is referred as social jetlag. The overall aim of this study is to test whether there is an association between both screen time and nighttime texting and social jetlag among 13- to 15-year-old adolescents in Sweden. This study included a cross-sectional survey in which data were collected from all schools with grades 7 and 8 in four municipalities in southern Sweden. The sample consisted of 1518 students (72.7% response rate), among whom 50.7% were girls. Ages varied between 13 and 15 years (mean, 13.9; standard deviation (SD), 0.4). Social jetlag was defined as more than 2 h difference between bedtime and wake-up time on school days compared to weekends. The prevalence of social jetlag among this study population was 53.9%. After adjusting for age, sex, and economic status, the multivariate binary logistic regression analysis results showed that increased screen time (*p* < 0.001) and texting at night (*p* = 0.002) were significantly associated with social jetlag. Irregular bedtime and wake-up habits on school days and weekends are associated with nighttime texting and increased screen time. For future research, more focus should be given to identifying causality factors and gain an understanding of the effects of social jetlag, which will help in developing appropriate public health messages and intervention programs.

## Introduction

The notion of adolescents as a genuinely healthy age group has contributed to them being neglected in public health research (Gore et al., 2011). Measuring adolescent health requires sound information, including social and environmental determinants (Patton et al., 2010). Adolescence and young adulthood are unique periods of life, and investments in adolescent health will contribute to a healthier start in life (Patton et al., 2016). Recent estimates suggest that one in five children and young adults have difficulties sleeping (Calhoun et al., 2014). There is a rapidly rising prevalence of sleep problems among adolescents, which is associated with poor functioning (Langberg et al., 2017). Therefore, the increasing prevalence of sleep problems has become a recognized international public health problem (Gradisar et al., 2011). Among children, insufficient sleep increases the risk of neuropsychological complications (Gregory et al., 2009). A literature review, including 36 papers on the relationship between sleep and electronic media among adolescents has concluded that delayed bedtime and shorter total sleep time are consistently related to media use (Cain and Gradisar, 2010). Television viewing, use of the computer, internet, and electronic gaming, the use of mobile phones, and listening to music were identified as having a negative impact on sleep (both delayed and shortened sleep time) among adolescents (Cain and Gradisar, 2010). A study on sleep quality during early childhood found that independent use of different electronic media now begins at less than 3 years old, which is before the children have learned to read (Genuneit et al., 2018). Children learn to use electronic devices on their own, which are handed to them by their parents, resulting in a significant amount of screen time (from computer, television, and mobile device use) (Kabali et al., 2015). The frequent use of electronic media is generating a debate about to what extent this might have an adverse effect on health (Milde-Busch et al., 2010). Therefore, today’s children are exposed to a greater amount of electronic media than ever before, which now requires constructive and in-depth research on how this affects their physiological and psychological well-being. Here, we investigated irregular sleep (“social jetlag”), screen time and nighttime texting among adolescents.

Humans have 24-h biological rhythms (referred to as circadian rhythms). Mammals express multiple biological rhythms structured into multiple complex interactions (Touitou and Haus, 2012). In humans, circadian rhythms are driven by endogenous (genetics) and exogenous (e.g., social life, light-dark cycle, and sleep-wake cycle) factors (Touitou and Haus, 2012). The internal time-keeping system or biological clock is represented by the suprachiasmatic nuclei (SCN) of the anterior hypothalamus, which controls the circadian system (Oginska and Pokorski, 2006; Touitou and Haus, 2012). Circadian rhythm refers to our daily synchronized rhythm, which is also known as our 24-h biological clock (Oginska and Pokorski, 2006; Touitou and Haus, 2012). Thus, a temporal dissociation can occur when the biological clock is not synchronized with the astronomical clock because of other factors, such as jetlag, night work, shift work, mood disorder, and the use of certain medications (Touitou et al., 1990; Reinberg and Ashkenazi, 2008). Sleep has a bidirectional relationship with the circadian system as not only insufficient sleep but also mistimed sleep that interrupts biological clock has an impact on the circadian system (Kryger et al., 2011). The discrepancy between social and biological clock due to sleep and wake up time difference across weekdays and weekends is referred as social jetlag (Wittmann et al., 2006). Social jetlag affects almost all of us throughout life, especially young adults (Wittmann et al., 2006). Today’s adolescents and young people are at an increased risk of social jetlag because of electronic media habits (Cain and Gradisar, 2010). The negative effects on sleep (both delayed and shortened) because of inappropriate electronic media use among adolescents and young people is alarming (Cain and Gradisar, 2010).

Insufficient sleep among adolescents is significantly associated with negative physiological consequences, such as obesity, depression, anxiety, mood disturbance, suicidal ideation, and drug and alcohol use (Gupta et al., 2002; Chen et al., 2008; Stoner et al., 2018). Adolescents with poor sleep quality and decreased sleep duration tend to have a lower sense of well-being, poor academic results, and a decreased quality of life (Wittmann et al., 2006; Baert et al., 2015; Johansson et al., 2016). Moreover, chronic sleep loss in adolescents is associated with poor judgment and risk-taking behaviors, such as drinking and driving, smoking, and substance abuse (Calamaro et al., 2009; Catrett and Gaultney, 2009). In studies among adolescents, social jetlag has been found to be associated with an unhealthy dietary pattern, depressive symptoms, and multiple metabolic risks (Levandovski et al., 2011; Wong et al., 2015; Koopman et al., 2017; Almoosawi et al., 2018; Mota et al., 2019). Surprisingly, female participants with social jetlag had more depressive symptoms compared to men (Mathew et al., 2019).

For the development of adolescent health, sleep is a key element, and sleep (both psychologically and physiologically) is important (Touitou, 2013). However, the prevalence rate of sleep disorders is comparatively high among western countries, where the cumulative sleep debt has been associated with behavioral problems, poor school achievements, and fatigue (Touitou, 2013). Thus, the effects on sleep have been the object of many studies addressing the permanent social jetlag that is experienced by many adolescents, and this should be considered to be a matter of concern in public health (Touitou, 2013; Johansson et al., 2016).

Many variables have been investigated, although delayed bedtime and shorter total sleep time have been found to be most consistently related to electronic media use and nighttime texting with negative consequences (Gradisar et al., 2011; Pecor et al., 2016). Therefore, how electronic media use is associated with sleep and social jetlag among adolescents requires further study (Waterhouse et al., 2005).

## Purpose

The overall aim of this study is to address the research question of whether there is an association between social jetlag and screen time and nighttime texting among adolescents aged 13–15 in Sweden. To address this aim, we opted for a cross-sectional study where the following research question is examined: Is there any association between social jetlag, screen time, and nighttime texting among adolescents?

The specific objectives are:

(1) To examine the associations between experienced social jetlag and the level of daily screen time among adolescents; and
(2) To examine the association between social jetlag and the frequency of nighttime texting among adolescents.

## Materials and Methods

This study was conducted using the quantitative data from a larger research project (ISRCTN17006300) that was performed as a cross-sectional survey that collected data from all schools with grades 7 and 8 in four municipalities in southern Sweden. Respondents came from schools that were located in both urban and rural areas and from public as well as private schools. The sample consists of 1518 students (72.7% response rate), of whom 50.7% were girls. Ages varied between 13 and 15 years (mean, 13.9; standard deviation (SD), 0.4).

### Ethical Considerations

Guardians and students were informed in writing about the purpose of the study and that it was voluntary to participate in the study. Written informed consent was obtained. The students were told that there were no correct or incorrect answers. The study was approved by the Regional Ethical Review Board in Lund, EPN 2015/113. All procedures were conducted in accordance with the Declaration of Helsinki.

### Data Collection

In this cross-sectional study, each student in the four included municipalities in southern Sweden was invited to participate in this study. A survey questionnaire was distributed by the respective school nurses in connection with mandatory health interview (during school hours) in grades 7 and 8. The questionnaire was completed using paper and a pencil, and the school nurse was present to answer any questions related to the questionnaire.

### Questionnaire

The survey was based on the Sleep and Media Habits Questionnaire (SMHQ), which is designed to track sleep and screen habits in school-aged children (Garmy et al., 2012a). The SMHQ consists of questions about the time (hours and minutes) spent in front of a TV/computer per day that is not related to schoolwork, as well as sleep duration (hours and minutes) on nights before school days as well as on weekends, time for going to bed and waking up on school days and weekends, tiredness in school (never, rarely, often, and every day), and the frequency of texting/other messages (e.g., Facebook and Instagram) at night (never, a few times a year, sometimes every month, sometimes every week) during the school week.

### Description of Variables

#### Demographic Variables

Sex, age, and economic situation variables were considered to be the demographic information in this study. Age was a continuous variable, while sex was a dichotomized variable. The economic situation (self-assessed) variable had six options to answer, and, from those six options, a dichotomous variable was computed (very good or quite good economic situation/average or worse economic condition).

#### Social Jetlag

The major outcome variable in this study is social jetlag and it is defined and measured according to the theory and process presented by Wittmann et al. (2006). In this study, 93% of the participants had a sleep midpoint difference greater than or equal to 1 h. Thus, this study concluded that a midpoint difference of greater than or equal to 2 h is the social jetlag cut-off point, and it was coded as a dichotomized variable.

#### Screen Time

TV time and computer time (hours per day) were treated as continuous variables in the study. Total screen time was calculated based on the time spent using TV and computers. This continuous variable is categorized into three categorical variables of more or less than 2 h of screen time, more or less than 3 h of screen time and, more or less than 4 h of screen time.

#### Nighttime Texting

Another categorized variable is nighttime texting. The frequency of sending or receiving SMS/other messages (e.g., Facebook, Instagram, and Snap) during the night had four response options (never, yearly, monthly, and weekly), and a dichotomized variable for texting habit was generated (weekly/less than weekly).

### Data Analysis

Descriptive and analytical statistics have been conducted. Data analysis is conducted using the SPSS version 24 (IBM Corp., IBM SPSS Statistics for Windows). The mean, median, and percentages of the descriptive statistics are presented for the continuous variables, and the frequencies with percentages are presented for the categorical variables. Bivariate analyses with Pearson Chi-square test were performed to observe the *p*-value between dependent and independent variables. Binary logistic regression was used to analyze the confidence interval (CI) and the odds ratio (OR) was used to represent the data. A *p*-value <0.05 was considered to be statistically significant. In the crude analysis, the association between categorized social jetlag (more than 2 h was determined to indicate the presence of social jetlag) and screen time, texting at night, and demographic variables were investigated using binary logistic regression analysis. Multivariate binary logistic regression was used to analyze the adjusted OR between the dependent and independent variables, thus controlling for the possible effect modifiers and potential confounders in the binary logistic regression models. Different kinds of electronic use and social jetlag have been shown to be significantly affected by socio-economic status, age, and sex in earlier studies (Thomas et al., 2010; Short and Louca, 2015; Komada et al., 2019; Mathew et al., 2019). Therefore, in this study, these three factors were adjusted in the multivariate binary logistic regression analysis model.

## Results

### Sample Characteristics

The population sample consisted of approximately an equal number of male (49.3%) and female (50.7%) participants among the 1518 study participants. The mean participant age was 13.89 years, and the range was 13–15 years. For the self-reported economic situation, 1227 (80.8%) of the participants described their economic situation as good or very good (Table 1). Among the participants who answered the questions about sleep (*n* = 1425, 93.9%), 53.9% (*n* = 818) experienced social jetlag (a sleep midpoint difference greater than or equal to 2 h). Among the participants (*n* = 1354, 89.9%), the total screen time for using a computer and television was over 4 h for 36% (*n* = 491) of the participants, and males comprised 57.2% (*n* = 281) of these participants. Among the participants who reported on their nighttime texting habit (*n* = 1510, 99.5%), 37.6% (*n* = 568) reported never texting at night. A nighttime texting habit of sometimes every week was shown for 25.8% (*n* = 389) of these participants.

**TABLE 1:** Descriptive characteristics of the characteristics of the participants (*N* = 1518) from southern Sweden.

**Variables**	**Male n (%)**	**Female n (%)**	**Total n (%)**
**Age (*N* = 1518)**			
13 years	84 (5.5)	106 (7.0)	190 (12.5)
14 years	651 (42.9)	661 (43.5)	1312 (86.4)
15 years	13 (0.9)	3 (0.2)	16 (1.1)
Missing Data	–	–	–
**Socio-economic situation (*N* = 1518)**			
Very good or good economic situation	602 (39.7)	625 (41.2)	1227 (80.8)
Average or worse economic situation	146 (9.6)	145 (9.6)	291 (19.2)
Missing Data	–	–	–

**Continuous variable**	**Mean**	**Mean**	**Mean**
	**(hr:min) (SD)**	**(hr:min) (SD)**	**(hr:min) (SD)**

**Bedtime**			
Weekdays	22.20 (0.89)	22.15 (0.83)	22.20 (0.86)
Weekends	24.10 (1.40)	23.45 (1.24)	23.59 (1.34)
**Wake up time**			
Weekdays	6.41 (0.46)	6.58 (0.48)	6.52 (0.48)
Weekends	10.05 (1.43)	9.42 (1.19)	9.55 (1.33)
**Sleep Duration**			
Weekdays	8.13 (0.96)	8.01 (0.98)	8.07 (0.97)
Weekends	9.67 (1.31)	9.71 (1.14)	9.69 (1.23)
Screen time	3.72 (2.14)	3.06 (2.06)	3.38 (2.12)

**Categorical variable**	**n (%)**	**n (%)**	**n (%)**

**Screen time (4 h cutoff)**			
Less than 4 h	375(57.2%)	498(70.3%)	873(64.0%)
4 h or more	281(42.8%)	210(29.7%)	491(36.0%)
**Night time texting**			
Less than weekly	419(75.2%)	445(78.9%)	864(77.1%)
Weekly	138(24.8%)	119(21.1%)	257(22.9%)

### Bivariate Analysis (Pearson Chi-Square Test)

The bivariate analysis was performed in this study between the dependent variable social jetlag and the independent variables such as the demographic variables (Table 2). From the demographic variables of age, sex, and economic situation, only the participant’s sex (*p* = 0.001) showed a statistically significant association with social jetlag. However, social jetlag is statistically significantly associated with nighttime texting (*p* = 0.001) and screen time [>2 h of screen time (*p* = 0.025), >3 h of screen time (*p* = 0.002), and >4 h of screen time (*p* < 0.001)]. Because the category for >4 h of screen time showed the highest significance in the analysis, this category was used for further analysis. Average sleep duration was not associated with social jetlag (*p* = 0.191).

**TABLE 2:** Descriptive characteristics from the Chi-square test on association of social jetlag and independent variables of adolescent participants (*N* = 1518) from southern Sweden.

**Variables**	**Social jetlag**	**Social jetlag**	**Total n**	***P* value**
	**No n (%)**	**Yes n (%)**		
**Age (*N* = 1425)**				
13 years	67 (37.9)	110 (62.1)	177	–
14 years	536 (43.4)	699 (56.6)	1235	0.259
15 years	4 (0.3)	9 (0.6)	13	–
**Economic situation (*N* = 1425)**				
Very good or good economic situation	499 (43.2)	657 (56.8)	1156	0.367
Average or worse economic situation	108 (40.1)	161 (59.9)	269	–
**Sex (*N* = 1425)**				
Male	264 (38.3)	426 (61.7)	690	0.001*
Female	343 (46.7)	392 (53.3)	735	–
**Nighttime texting (*N* = 1057)**				
Less than Weekly	400 (49.0)	417 (51.0)	817	–
Weekly	88 (36.7)	152 (63.3)	240	0.001*
**Screen time (*N* = 1290)**				
Less than 4 h of screen time	390 (47.0)	440 (53.0)	830	
4 h or more of screen time	161 (35.0)	299 (65.0)	460	<0.001*

*Statistically significant (*p* < 0.05).

### Crude Analysis: Binary Logistic Regression Analysis

In Table 3, the crude analysis between social jetlag and independent variables for the unadjusted OR and a 95% CI has been presented with the sex-segregated value. The sex of the participants showed a significant OR (OR = 1.404), which means that male participants were 1.4 times more likely to experience social jetlag in this study. Nighttime texting also showed a significant OR (OR = 1.487), meaning that texting often at night was 1.5 times more likely to be associated with social jetlag. Four hours or more of screen time was also significant, indicating that screen time is 1.5 times more likely to be associated with social jetlag (OR = 1.547). However, age and economic situation did not show any significance in this crude analysis. Because the sex of the participants was significant in both bivariate and crude analyses for the adjusted analysis, the sex-segregated analysis is included.

**TABLE 3:** Sex-segregated crude analysis of the association between social jetlag and independent variables among 13–15 year old adolescents in southern Sweden.

**Variables**	**Male**	**Female**	**Total**
	**OR (CI 95%)**	**OR (CI 95%)**	**OR (CI 95%)**
**Age**			
13 years	1	1	1
14 years	0.658 (0.393–1.100)	0.875 (0.572–1.338)	0.710 (0.465–1.086)
15 years	0.639 (0.165–2.479)	(–)	0.912 (0.198–4.193)
**Economic situation**			
Very good or good economic situation	1	1	1
Average or worse economic situation	0.872 (0.592–1.283)	1.438 (0.985–2.099)	1.113 (0.775–1.598)
**Sex**			
Female	N/A	N/A	1
Male	N/A	N/A	1.404 (1.063–1.854)*
**Nighttime texting**			
Less than weekly	1	1	1
Weekly	1.375 (0.906–2.086)	1.916 (1.253–2.928)	1.487 (1.072–2.062)*
**Screen time**			
Less than 4 h of screen time	1	1	1
4 h or more of screen time	1.475 (1.056–2.060)	1.702 (1.214–2.385)	1.547 (1.155–2.072)*

*Statistically significant (*p* < 0.05). (–) The number of the respondents was too low to show the result.

### Adjusted Analysis: Multivariate Binary Logistic Regression Analysis

The adjusted analysis is presented to determine the association between dependent and independent variables in sex-segregated and total data that was adjusting for age, sex, and socio-economic status (Table 4). After adjusting for age sex and economic status, screen time (*p* < 0.001) was statistically significantly associated with social jetlag. In Table 4, the adjusted analysis is presented to determine the association between dependent and independent variables in overall and sex-segregated data adjusting for age, sex, and socio-economic status. After adjusting for age, sex, and economic status, texting at night (*p* = 0.002) was statistically significantly associated with social jetlag. According to the bivariate and crude analysis, it is assumed that sex can be a potential cofounder. However, in the multivariate regression model with and without the variable sex in the model, the effect size was not significantly affected, and thus, sex was not a confounding factor in this regression model.

**TABLE 4:** Multivariate logistic regression analysis between social jetlag with screen time and nighttime texting adjusting by age sex and economic status.

**Variables**	**Male participants**		**Female participants**		**Total sample**	
	**OR (95% CI)**	***P***	**OR (95% CI)**	***P***	**OR (95% CI)**	***P***
**Total screen time**						
Less than 4 h of screen time	Ref	Ref	Ref	Ref	Ref	Ref
4 h or more of screen time	1.534 (1.095–2.146)^AE^	0.013	1.691 (1.205–2.373)^AE^	0.002*	1.596 (1.258–2.025)^ASE^	<0.001*
**Nighttime texting**						
Less than weekly	Ref	Ref	Ref	Ref	Ref	Ref
Weekly	1.386 (0.912–2.107)^AE^	0.127	1.857 (1.210–2.848)^AE^	0.005*	1.609 (1.193–2.170)^ASE^	0.002*

*Statistically significant (*p* < 0.05). ^*A*^Adjusted for age. ^*S*^Adjusted for sex. ^*E*^Adjusted for socio-economic status.

## Discussion

Here, we report a statistically significant association between screen time and social jetlag among adolescents. This result is similar to other studies where screen time was negatively associated with sleep habits (Calamaro et al., 2009; Cain and Gradisar, 2010). A study conducted in the United States has shown an extreme prevalence of bedtime technology use and that this is strongly associated with sleep-related complications (Gradisar et al., 2013). This study claimed that nine out of ten Americans have reported using a technological device before sleeping and that the more interactive the device is (e.g., computer, laptop, cell phone, or video console), the greater the risk of reported sleep problems, including problems sleeping and unrefreshing sleep. Moreover, that study found that watching TV is popular (60% of the total population) before going to sleep (Gradisar et al., 2013). In a systematic review paper that assessed the scientific literature among school-aged children and adolescents regarding the association between screen time and sleep outcomes, 90% of the 67 articles included in the study claimed that screen time is adversely associated with sleep outcomes among adolescents (Hale and Guan, 2015).

A study conducted in the United States suggested that technological device use before sleep is highly prevalent and this is also associated with sleep problems; 72% of the participating adolescents reported using a cell phone before going to sleep (Gradisar et al., 2013). In this study, we found similar results, that night time texting use of cell phones is significantly associated with social jetlag (Gradisar et al., 2013). Studies have identified a statistically significant association between social jetlag and health-related disorders, and they also showed that irregular sleep timing is associated with an unhealthy lifestyle (Roenneberg et al., 2019). Additionally, social jetlag and its association with adverse health outcomes are often studied as a sleep discrepancy, and there is currently no consensus on outcomes (Roenneberg et al., 2019). Therefore, in social jetlag studies, final outcomes are often described in terms of sleep habits. Here, we report a high prevalence of social jetlag and its association with screen time and nighttime texting. Similarly, other studies addressing adolescents and their sleep behavior showed that the use of multiple electronic media was associated with sleep.

In this study, we found a high prevalence of social jetlag, which suggests the need for further studies and the development of intervention/prevention strategies. In a cohort study, 43,880 subjects were followed for 13 years to address any possible association between mortality and both weekday and weekend sleep (Åkerstedt et al., 2019). The authors reported a significant association between mortality and weekday and weekend sleep (Åkerstedt et al., 2019). Short sleep duration during on weekdays had a higher mortality rate if there was no compensation with more sleep on the weekends (Åkerstedt et al., 2019). Here, we concluded that there is a high prevalence of social jetlag and that the study population compensates for their short sleep duration during the weekdays by sleeping more during the weekends. However, social jetlag is often not studied among adolescents, but it is also important to remember that social jetlag is something that affects most people at some point in their life, although adolescents are at relatively greater risk (Wittmann et al., 2006). In a study on sleep behavior among adolescents, after controlling for different emotional behaviors, nighttime use of any electronic device was associated with poorer sleep quality (Woods and Scott, 2016). Nighttime sleep and daytime functioning of adolescents are affected by technology use before sleep (Johansson et al., 2016). Similar studies have reported how nighttime texting habits are associated with disrupted sleep (Garmy and Ward, 2018) and that screen time (television and computer) is associated with sleep behavior (Garmy et al., 2012b) among young people.

### Strengths and Limitations

This study focused on one of the newest areas of social epidemiology: adolescent health and technological behavior. Few studies have investigated electronic media use and its impact on social jetlag among adolescents.

This paper has emphasized adolescents and nighttime texting, acknowledging that it has an impact on young people, which is well-known but under-investigated. Moreover, as a cross-sectional study, it is a representative sample of the total population and it had a relatively high participation rate, which is a strength in this study.

In this study, the main aim was to investigate whether the association of inconsistent sleep during weekdays and weekends, known as social jetlag, is associated with screen time and nighttime texting. However, one of the main limitations of this study was that the cross-sectional study design. Thus, this study could not determine a causal relationship between the dependent and independent variables. Moreover, there is no data regarding screen time difference for weekdays and weekends. Another limitation is that this study does not include any data on chronotype which could include a significant aspect in the study. Lastly, the data on night-time texting the frequency of texting has low resolution and does not cover more frequent options.

### Implications for Future Research

An in-depth literature review in this arena also showed a lack of research in developing countries. Although there has been some research on social jetlag focusing on ethnic minorities and social jetlag (Anothaisintawee et al., 2018), there have been no significant studies conducted in the developing countries, which should be addressed in future work. However, in developing countries, such research is complicated because electronic media use can be a potential confounding factor for social jetlag. In any future study regarding electronic media use, there should be a greater focus on the influence of social jetlag on eating behavior, composition of the daily diet, and mealtime among different age groups (Mota et al., 2019). Moreover, a detailed study including depressive symptoms and substance use (e.g., coffee or energy beverage consumption) should be included (Chen et al., 2008; Calamaro et al., 2009; Cain and Gradisar, 2010; Pecor et al., 2016). This study has focused on the association, and future studies on this topic should address the clinical significance, the magnitude of the association, and the causality of social jetlag. Future research requires a methodological approach to addressing the causal pathways between screen time, nighttime texting, and sleep. Future research should also focus on more characteristics related to screen-use that can potentially cause behavioral changes, such as timing, duration, screen size, volume, and closeness to face (Åkerstedt et al., 2019). In conclusion, future research needs to develop and measure the impact and magnitude of this problem to generate youth-appropriate public health messaging and interventions that will reduce the risk of screen time and nighttime texting before or during bed and their potential consequence on sleep, health, and well-being.

## Data Availability Statement

The datasets generated for this study are available on request to the corresponding author.

## Ethics Statement

The studies involving human participants were reviewed and approved by the Regional Ethical Review Board in Lund, EPN 2015/113. Written informed consent to participate in this study was provided by the participants’ legal guardian/next of kin.

## Author Contributions

PG designed the research. MH and PG analyzed the data. MH wrote the first draft of the manuscript. MH and PG revised the manuscript and approved the final version.

## Conflict of Interest

The authors declare that the research was conducted in the absence of any commercial or financial relationships that could be construed as a potential conflict of interest.
